# A chromosome-level genome assembly of the darkbarbel catfish *Pelteobagrus vachelli*

**DOI:** 10.1038/s41597-023-02509-0

**Published:** 2023-09-08

**Authors:** Gaorui Gong, Wensi Ke, Qian Liao, Yang Xiong, Jingqi Hu, Jie Mei

**Affiliations:** Hubei Hongshan Laboratory, College of Fisheries, Huazhong Agricultural University, Wuhan, 430070 China

**Keywords:** Genomics, Genome

## Abstract

The darkbarbel catfish (*Pelteobagrus vachelli*), an economically important aquaculture species in China, is extensively employed in hybrid yellow catfish production due to its superior growth rate. However, information on its genome has been limited, constraining further genetic studies and breeding programs. Leveraging the power of PacBio long-read sequencing and Hi-C technologies, we present a high-quality, chromosome-level genome assembly for the darkbarbel catfish. The resulting assembly spans 692.10 Mb, with an impressive 99.9% distribution over 26 chromosomes. The contig N50 and scaffold N50 are 13.30 Mb and 27.55 Mb, respectively. The genome is predicted to contain 22,109 protein-coding genes, with 96.1% having functional annotations. Repeat elements account for approximately 35.79% of the genomic landscape. The completeness of darkbarbel catfish genome assembly is highlighted by a BUSCO score of 99.07%. This high-quality genome assembly provides a critical resource for future hybrid catfish breeding, comparative genomics, and evolutionary studies in catfish and other related species.

## Background & Summary

Siluriformes, better known as catfishes, constitute a significant portion of the teleost orders, making up approximately 11% of all species. With 39 families and around 4,094 species documented to date, this order is among the largest in existence^1^. Owing to their delectable meat, minimal intermuscular bone, and impressive feed conversion ratio, catfishes have risen to become one of the top three farmed fish and shellfish, boasting a production of 5,519 kilotons in 2017 alone^2,3^.

Yellow catfish (*Pelteobagrus fulvidraco*) is an important aquaculture fish species in China, the production of which was about 565 thousand tons in 2020^4^. Darkbarbel catfish (*Pelteobagrus vachelli*), a close relative of yellow catfish within the family Bagridae, has a faster growth rate than yellow catfish^5^. Recently, hybrid yellow catfish (*P*. *fulvidraco*♀ × *P*. *vachelli*♂) has been widely cultured in China due to its faster growth rate and better emergence rate^6^. Unfortunately, specific research on the darkbarbel catfish genome proved difficult to access, but the general principles of genomics in aquaculture suggest that having a high-quality reference genome for this species will have significant benefits for breeding programs and other genetic studies. The insights gained from the darkbarbel catfish genome can also support the successful production of the hybrid yellow catfish, by contributing to a better understanding of their genetic makeup and the genetic factors influencing their advantageous traits.

In this research, we have employed a combination of PacBio long-read sequencing and Hi-C technology to generate a high-quality, chromosome-level assembly of the darkbarbel catfish genome. With the development of this high-quality reference genome, we foresee a significant propulsion in the field of population genetics and the identification of functional genes associated with critical economic traits in the darkbarbel catfish. The elucidation of these genomic underpinnings is expected to provide deeper insights into the hybrid vigor observed in catfish hybrids, thereby contributing to the optimization of hybrid breeding strategies.

## Methods

### Sample collection and sequencing

An adult female darkbarbel catfish was collected from the Yangtze River in Wuhan, Hubei, China. High-molecular weight (HMW) genomic DNA was extracted from muscle for Illumina sequencing and PacBio SMRT sequencing. The quality and quantity of the extracted DNA was assessed using standard agarose gel electrophoresis and a Qubit fluorometer (Thermo Fisher Scientific, USA).

For Illumina sequencing, the genomic DNA was randomly sheared to ~350 bp fragments, and a paired-end genomic library was prepared following the manufacturer’s protocol. Then, the library was sequenced on an Illumina HiSeq X-Ten platform using a paired-end 150 bp layout. For PacBio sequencing, the genomic DNA was used to construct SMRTbell libraries following the manufacturer’s protocol. After that, the libraries were sequencing on a PacBio Sequel platform with SMRT technology. Finally, we generated 96.33 Gb Illumina short-read data and 109.76 Gb of raw PacBio continuous long reads (CLR) with an average read length of 13.8 kb and a N50 read length of 22.4 kb (Table 1).

**Table 1:** Statistics of the sequencing data.

Library type	Platform	Tissue	Data size (Gb)	Average length (bp)
WGS short reads	Illumina HiSeq X-Ten	Muscle	96.33	150
WGS long reads	Pacbio Sequel II	Muscle	109.76	13,806
Hi-C	Illumina HiSeq X-Ten	Blood	89.12	150
RNA-Seq	Illumina HiSeq X-Ten	Spleen, kidney, brain, muscle, ovary, and liver	13.11	150

For genome scaffolding, a Hi-C library was prepared using blood sample from the same darkbarbel catfish used for genomic DNA sequencing. The Hi-C library construction, including cell crosslinking, cell lysis, chromatin digestion, biotin labelling, proximal chromatin DNA ligation and DNA purification, was performed as previously described^7^, and the resulting Hi-C library was then subjected to paired-end sequencing with 150 bp read lengths on an Illumina HiSeq X-Ten platform. As a result, 89.12 Gb of Hi-C read data was generated (Table 1).

To aid in genome annotation, total RNA was extracted from multiple tissues, including spleen, kidney, brain, muscle, ovary and liver, and the quality were evaluated using a NanoDrop 2000 spectrophotometer (Thermo Fisher Scientific, USA) and an Agilent 2100 Bioanalyzer (Agilent Technologies, USA). The mixed RNA sample was used to construct a cDNA library using the TruSeq Stranded mRNA Library Prep Kit (Illumina, USA) following the manufacturer’s protocol. The library was then sequenced on an Illumina HiSeq X-Ten platform using a paired-end 150 bp layout, and 13.11 Gb of data was obtained (Table 1).

### Genome assembly and polishing

**Fig. 1:**
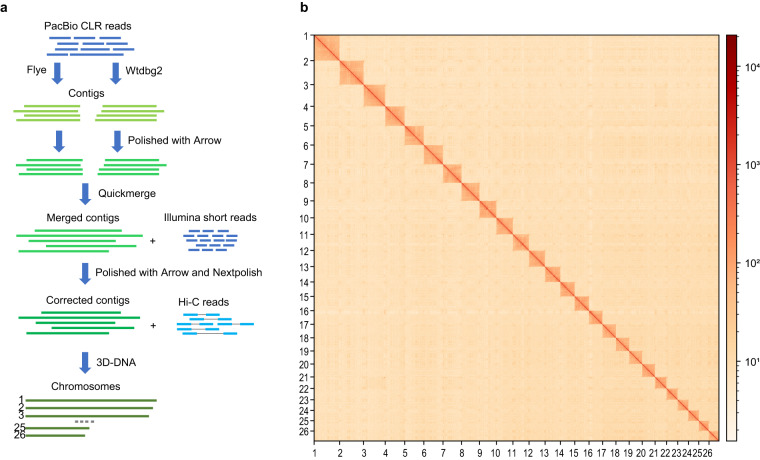
Overview of the genome assembly for darkbarbel catfish. (**a**) Flow chart illustrating the genome assembly pipeline for darkbarbel catfish. (**b**) Hi-C heatmap (200-kb resolution) showcasing the interaction frequencies between different chromosomes of darkbarbel catfish.

To assemble the genome, we utilized two different assemblers: Wtdbg2 v2.5^8^ and Flye v2.9^9^ (Fig. 1a). The assembly generated by each assembler with default parameters was then polished using Arrow, a consensus algorithm that can generate highly accurate consensus sequences from PacBio subreads. The two polished assemblies were then merged using Quickmerge^10^, a tool that combine multiple genome assemblies into a single consensus assembly. The resulting merged assembly was then polished twice using two rounds of Arrow and two rounds of NextPolish with default parameters (Fig. 1a). We used PacBio subreads for Arrow and Illumina short reads for NextPolish. The resulting assembly consists of 318 contigs and has a total length of 691.96 Mb (Table 2).

**Table 2:** Assembly statistics of darkbarbel catfish.

Type	Contig	Scaffold
Number	368	77
N50 (Mb)	13.30	27.55
L50	21	11
Max length (Mb)	25.86	43.66
Total length (Mb)	691.96	692.10

### Hi-C scaffolding

The raw Hi-C reads were processed to remove adapters and low-quality bases using Fastp v0.20.1^11^ with parameters -q 20 -l 50. The processed reads were then aligned to the assembly using the Juicer pipeline^12^. Then the 3D-DNA pipeline^13^ was used to group the contigs into chromosomes, orient and order the contigs within each chromosome. To further improve the quality of the assembly, we manually corrected the errors using the Juicebox Assembly Tools^12^. Following the scaffolding procedure, 691.13 Mb were successfully anchored to the 26 chromosomes (Fig. 1b), encompassing an impressive 99.9% of the total assembly size of 692.10 Mb (Table 2). The observed chromosome number concurs with the karyotype analysis reported in the previous study^14^. The scaffold N50 reached a substantial 27.55 Mb for the final assembly (Table 2). Notably, among the 26 chromosomes, 16 of them exhibited exceptional contiguity with no more than 10 gaps observed (Table 3).

**Table 3:** Assembly statistics for chromosomes.

Name	Length (bp)	Gaps
Chromosome 1	43,657,716	13
Chromosome 2	40,896,051	14
Chromosome 3	36,888,217	27
Chromosome 4	33,276,060	5
Chromosome 5	32,707,000	6
Chromosome 6	32,597,123	16
Chromosome 7	31,818,857	42
Chromosome 8	30,665,500	13
Chromosome 9	28,731,355	6
Chromosome 10	28,256,991	15
Chromosome 11	27,553,930	4
Chromosome 12	27,443,794	21
Chromosome 13	26,028,991	1
Chromosome 14	24,604,000	8
Chromosome 15	24,378,500	7
Chromosome 16	23,162,500	35
Chromosome 17	22,852,063	2
Chromosome 18	22,476,563	7
Chromosome 19	22,294,437	3
Chromosome 20	22,045,500	3
Chromosome 21	19,572,866	18
Chromosome 22	19,205,148	1
Chromosome 23	18,096,000	8
Chromosome 24	17,657,601	5
Chromosome 25	17,528,722	1
Chromosome 26	17,005,764	10
Unplaced	703,650	0

### Repeat annotation

**Fig. 2:**
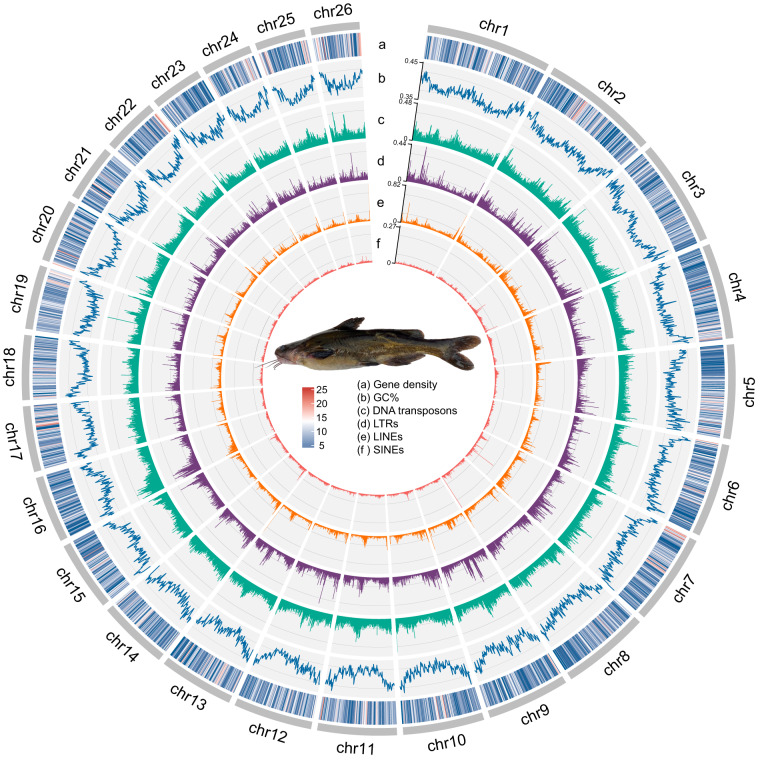
Genomic landscape of darkbarbel catfish. Circos plot of darkbarbel catfish illustrating from outside to inside, gene density (**a**), GC content (**b**), and the densities of DNA transposons (**c**), LTRs (**d**), LINEs (**e**), and SINEs (**f**), all represented in 200-kb genomic windows.

RepeatModeler v2.0.2^15^ firstly identified repetitive sequences in the genome assembly using several tools, including RECON, RepeatScout, TRF, Ltr_retriever, and LTRharvest. The identified sequences were then clustered and classified into families using RepeatModeler. The classified libraries were combined with the Teleostei library from Repbase^16^. RepeatMasker v4.1.4^17^ was performed to mask repetitive sequences in the genome assembly using the combined library generated by RepeatModeler. A significant portion of the genome, approximately 35.79%, is masked, resulting in 247,692,317 bp being identified as repetitive elements. Retroelements, including long terminal repeats (LTRs, 6.90%), long interspersed nuclear elements (LINEs, 5.87%), and short interspersed nuclear elements (SINEs, 1.01%), collectively comprise the largest proportion, occupying 13.77% of the genome (Fig. 2). Furthermore, DNA transposons occupy a notable 11.48% of the genome, while unclassified elements add a nuanced layer, constituting 4.69% of the genomic landscape (Fig. 2).

### Gene prediction and function assignment

In this research, we employed a comprehensive approach combining transcriptome-based, *de novo*, and homology-based methods to predict genes within the genome. For transcriptome-based prediction, RNA-seq reads underwent stringent quality filtering using Fastp v0.20.1, with specific parameters set at -q 20 -l 50. These filtered reads were then aligned to the genome assembly using HISAT2 v2.2.1^18^, followed by assembly using StringTie v2.2.1^19^. Gene structures were subsequently predicted utilizing TransDecoder v5.5.0 (https://github.com/TransDecoder/TransDecoder). For *de novo* prediction, RNA-seq aligned BAM files served as input for training the AUGUSTUS v3.4.0^20^ gene prediction tool via BRAKER^21^. This trained model was then employed to predict gene structures within the genome. In the homology-based prediction, we utilized miniport v0.11^22^ to align protein sequences from *P. fulvidraco*^7^, *Silurus meridionalis*^23^, and *Ictalurus punctatus*^24^ to the genome assembly, enabling the prediction of gene structures based on homologous evidence. To consolidate the results from these three methods, EvidenceModeler^25^ was employed, enabling the merging and integration of gene predictions. Following the gene prediction, the finalized gene sets derived from preceding methods underwent functional annotation through matching with a variety of databases. In particular, we utilized BLASTP v2.9.0^26^ to align the anticipated genes with SwissProt^27^, TrEMBL^28^, eggNOG^29^, and the NCBI non-redundant (NR) protein databases.

**Fig. 3:**
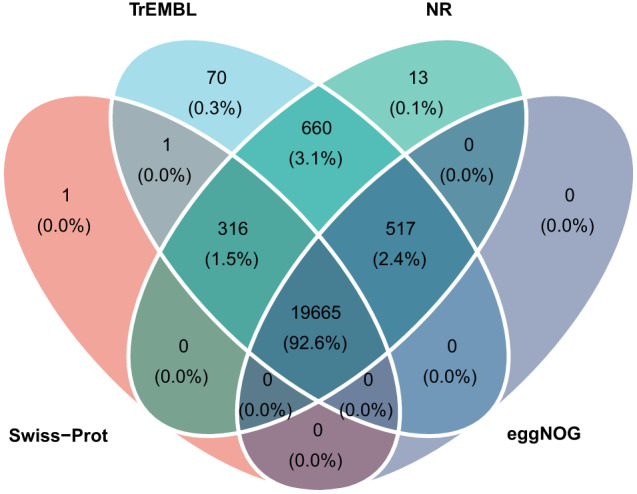
Venn diagram of function annotations from various databases. The Venn diagram displays the overlap and uniqueness of functional gene annotations derived from 4 databases: TrEMBL, NR, Siwss-Prot, and eggNOG.

In total, we successfully predicted 22,109 protein-coding genes within the genome (Fig. 2). These predicted genes displayed an average coding sequence length of 1,695.04 bp, an average gene length of ~15 kb, and an average exon number of 10. Further, 96.1% of the total predicted genes, which equates to 21,243 genes, were successfully assigned with at least one functional annotation (Table 4 and Fig. 3).

**Table 4:** Statistics of functional annotation result.

Database	Number
Swiss-Prot	19,983 (90.38%)
TrEMBL	21,229 (96.02%)
NR	21,171 (95.76%)
eggNOG	20,182 (91.28%)

### Genome synteny analysis

**Fig. 4:**
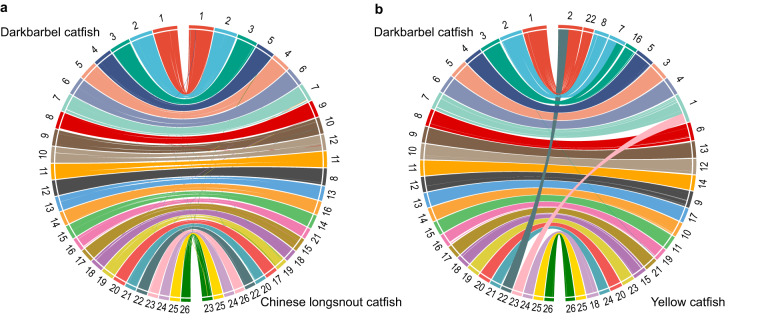
Chromosome sequence synteny comparisons. (**a**) Syntenic relationship between the darkbarbel catfish genome and the Chinese longsnout catfish genome. (**b**) Syntenic relationship between the darkbarbel catfish genome and the yellow catfish genome. Each line connects a pair of homologous sequences between the two species.

To compare the whole genome synteny, two chromosome-level genomes of Bagridae including yellow catfish^30^ and Chinese longsnout catfish (*Leiocassis longirostris*)^31^ were aligned to the genome assembly of darkbarbel catfish using LAST v1354^32^ with default parameters. The synteny were visualized using Circos v0.69.9^33^. A high degree of synteny conservation between the compared genomes was observed (Fig. 4).

## Data Records

All the raw sequencing data utilized in this study, including WGS, RNA-Seq, and Hi-C, have been deposited in the NCBI database under the BioProject accession number PRJNA819563. Specifically, the Illumina WGS data was archived with the accession number SRR24926343^34^, while the PacBio WGS data was deposited with the accession number SRR22354957^35^. The RNA-Seq and Hi-C data sets were archived under the accession numbers SRR24928263^36^ and SRR21799063^37^, respectively. The genome assembly is available for public access at the NCBI GenBank under the accession number GCA_030014155.1^38^. Genome annotations, along with predicted coding sequences and protein sequences, can be accessed through the Figshare^39^.

## Technical Validation

To evaluate the completeness of the genome assembly, we used the BUSCO v5.4.2^40^ with the Actinopterygii database (actinopterygii_odb10) to assess the presences of conserved sing-copy genes in the assembly. Out of the total 3,640 BUSCO groups searched, an impressive 99.07% were identified as complete, indicating a high level of gene content preservation. Among these, 98.54% were both complete and present as single-copy genes, further emphasizing the quality of the assembly. Additionally, only 0.33% of the BUSCOs were fragmented, and 0.60% were missing from the assembly (Table 5). This demonstrates the remarkable completeness and conservation of gene content in the darkbarbel catfish genome assembly, achieving one of the best BUSCO scores observed among reported catfish genomes.

**Table 5:** BUSCO assessment result.

Type	Number
Complete BUSCOs	3606 (99.07%)
Complete and single-copy BUSCOs	3587 (98.54%)
Complete and duplicated BUSCOs	19 (0.52%)
Fragmented BUSCOs	12 (0.33%)
Missing BUSCOs	22 (0.60%)
Total BUSCO groups searched	3,640

To ensure the quality and accuracy of the genome assembly, we employed a two-step validation process. Firstly, the assembly’s Quality Value (QV) was quantified using Merqury^41^, resulting in a QV score of 40.89, reflecting a high-grade assembly. Then, we mapped the raw sequencing data back to the assembly. For WGS short reads, we utilized BWA v0.7.17^42^, which resulted in a high mapping rate of 99.79%. For mapping RNA-Seq reads, we used HISAT2 v2.2.1 and achieved an overall mapping rate of 96.44%.

## Data Availability

No custom software codes were developed as part of this research. All bioinformatics tools and pipelines were executed following the manual and protocols provided by the respective software developers. The versions of the software used, along with their corresponding parameters, have been thoroughly described in the Methods section.
